# Personalized Nutrition in the Era of Digital Health: A New Frontier for Managing Diabetes and Obesity

**DOI:** 10.1002/fsn3.71006

**Published:** 2025-09-26

**Authors:** Muhammad Tayyab Arshad, M. K. M. Ali, Sammra Maqsood, Ali Ikram, Faiyaz Ahmed, A. I. Aljameel, Ammar AL‐Farga, Md. Sakhawot Hossain

**Affiliations:** ^1^ Functional Food and Nutrition Program, Center of Excellence in Functional Foods and Gastronomy, Faculty of Agro‐Industry Prince of Songkla University Hat Yai Thailand; ^2^ Department of Physics, College of Sciences Imam Mohammad Ibn Saud Islamic University (IMSIU) Riyadh Saudi Arabia; ^3^ National Institute of Food Science and Technology University of Agriculture Faisalabad Faisalabad Pakistan; ^4^ University Institute of Food Science and Technology The University of Lahore Lahore Pakistan; ^5^ Department of Basic Health Sciences, College of Applied Medical Sciences Qassim University Buraydah Saudi Arabia; ^6^ Department of Biochemistry, Faculty of Science University of Jeddah Jeddah Saudi Arabia; ^7^ Department of Nutrition and Food Technology Jashore University of Science and Technology Jashore Bangladesh; ^8^ Department of Nutrition and Food Engineering Daffodil International University Dhaka Bangladesh

**Keywords:** AI‐driven nutrition, metabolic health, nutrigenomics, obesity

## Abstract

The integration of digital health technologies with personalized nutrition offers a transformative approach for managing diabetes and obesity. This emerging paradigm extends beyond generic dietary recommendations by tailoring interventions based on genetic, epigenetic, microbiome, and real‐time metabolic data. Tools such as continuous glucose monitors (CGMs), artificial intelligence (AI)‐driven meal planning, and mobile health applications enable dynamic dietary adjustments and improved disease monitoring. Data privacy, cost disparities, and the need for robust clinical validation are challenges that remain to be overcome, even if there is a potential benefit. This review examines the synergy between digital health technologies and precision medicine by elucidating the potential of personalized nutrition in chronic disease management. Our literature review demonstrates that tailored diet programs can enhance metabolic well‐being and overcome theoretical and practical challenges to their widespread implementation.

## Graphical abstract

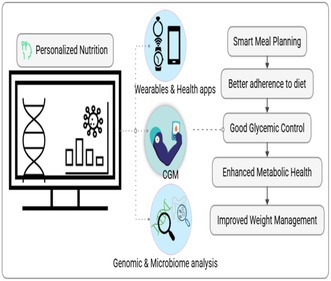

## 1 Introduction

The increasing worldwide prevalence of obesity and diabetes has rendered them two of the most significant public health issues in the modern era. Obesity is defined as abnormal or excessive accumulation of fat that represents a health hazard. Urbanization, nutritional transition, and physical inactivity are leading to rapid increases in the rates of obesity across low‐ and middle‐income countries (LMICs). Jaacks et al. (2019) reported that there have been a number of phases to the global obesity transition. More than 650 million adults are overweight, and by 2030, nearly 20% of the world's population could be affected (Blüher 2019; Alfaris et al. 2023).

Obesity causes metabolic syndrome, cardiovascular disease, and type 2 diabetes (T2D), leading to substantial health care expenses and economic burdens (Reilly et al. 2018; Seidell and Halberstadt 2015). Similarly, by 2021, 537 million adults will have diabetes, and this number is expected to rise to 783 million by 2045, making it a leading cause of death and disability (Sun et al. 2022). Neuropathy, retinopathy, and cardiovascular disease are exacerbated when nearly half of all diabetes cases remain undiagnosed, as stated by the International Diabetes Federation (IDF) (Cho et al. 2018; Saeedi et al. 2019). These risk factors are tightly linked with T2D, accounting for 90% of cases (Ogurtsova et al. 2017; Roglic 2016). According to Xu et al. (2017) and Li et al. (2020), lower‐income individuals are disproportionately affected by the differential socioeconomic variation in diabetes prevalence.

Traditional dietary recommendations have always been wide‐ranging, assuming that individuals' metabolism is equal (Hoevenaars et al. 2020). Traditional dietary pyramids and standardized calorie intake support this perspective. However, these traditional approaches do not consider the considerable inter‐individual variation in dietary responses due to genetic, microbiome, and metabolic variabilities. For example, genotype‐guided diets use genetic variations, such as FTO and TCF7L2, with obesity and glucose metabolism to personalize carbohydrate intake and improve diabetes management (Singar et al. 2024). Likewise, microbiome‐based nutrition considers diversity in gut microbiota; those with higher *Akkermansia muciniphila* levels can be anticipated to benefit the most from high‐fiber intake owing to enhanced short‐chain fatty acid production and improved insulin sensitivity (Gopal et al. 2024). These are merely a few of the many ways in which personalized nutrition breaks away from the one‐size‐fits‐all approach and moves towards individualized dietary intervention based on biology. There is increasing evidence that these recommendations do not work for most individuals because of variations in the gut microbiota, metabolic health, and genetics (Drabsch and Holzapfel 2019). If we consider low‐carbohydrate or Mediterranean‐style diets as examples, individuals might do better on them than on low‐fat diets (Wang and Hu 2018). Individualized nutrition plans that take into consideration each individual's specific biology and lifestyle are required because general dietary advice has not been successful in curbing the epidemic of obesity and diabetes (Moore 2020).

As Forster et al. (2016) explained, personalized nutrition is revolutionary because it employs genetic, epigenetic, and metabolic profiling to individualize dietary interventions instead of offering advice based on groups. Owing to recent progress in nutrigenomics, we currently know which genes are involved in how food acts on an individual to raise or lower the risk of developing diabetes and obesity (Mathers 2019). Conversely, wearable devices, artificial intelligence (AI)‐based meal planning apps, and continuous glucose monitors (CGMs) provide users with instant feedback, allowing them to adjust their nutrition plans in real time (Michel and Burbidge 2019; Celis‐Morales et al. 2017). Specifically, in diabetes prevention programs, digital health interventions such as gamification, nudges, and remote monitoring have been shown to enhance behavioral adherence (Partridge and Redfern 2018). Data privacy, accessibility, and clinical validation continue to be substantial hurdles toward broad implementation (Abrahams et al. 2019; O'connor et al. 2016). Notwithstanding such impediments, digital health intertwined with precision nutrition can revolutionize healthcare by addressing chronic diseases. It does this by introducing solutions that are more sustainable, effective, and customized than any of its predecessors (Johnson et al. 2021; Goetz and Schork 2018). The current investigation discovers a blend of digital health and personalized nutrition for diabetes and obesity care. It looks back at the current investigation, technological innovations, and potential future directions of this rapidly evolving topic.

### 1.1 Aim of the Review

With a focus on how practical it is for diabetes and obesity management, the investigation aims to explore the cumulative role of personalized nutrition in the information age. A break from traditional dietary advice and personalized nutrition offers personalized therapies by integrating genetic, epigenetic, and real‐time metabolic information. We believe that AI‐based meal planning, CGMs, and behavioral nudges have the potential to close the gaps in accessibility and clinical validation and simultaneously improve accuracy and compliance at the same time. Personalized nutrition is driven by scientific and technological advances such as nutrigenomics and microbiome analysis, as emphasized in this study. With regard to ethical concerns such as privacy protection and access, we balance the consequences of these developments for greater metabolic gains. This paper presents a complete picture of the promise and challenges of using personalized nutrition in disease prevention and treatment through the integration of recent evidence.

## 2 What Is Personalized Nutrition?

Based on the genetic makeup, gut microbiota, and live metabolic reactions of a person, customized nutrition calls for a transition from one‐size‐fits‐all dietary recommendations to personalized practices (Bashiardes et al. 2018; Drabsch and Holzapfel 2019). As persons are diverse in terms of gene expression (e.g., FTO, TCF7L2), gut microbiota (e.g., *A. muciniphila* ), and glucose tolerance, personalized nutrition differs from usual “one‐size‐fits‐all” practice (Zeevi et al. 2015; Kolodziejczyk et al. 2019). For example, nutrigenomic analysis can tell one who would familiarize themselves with losing weight and controlling diabetes on low‐glycemic or high‐protein diets (de Toro‐Martín et al. 2017; Ferguson et al. 2016).

**FIGURE 1:**
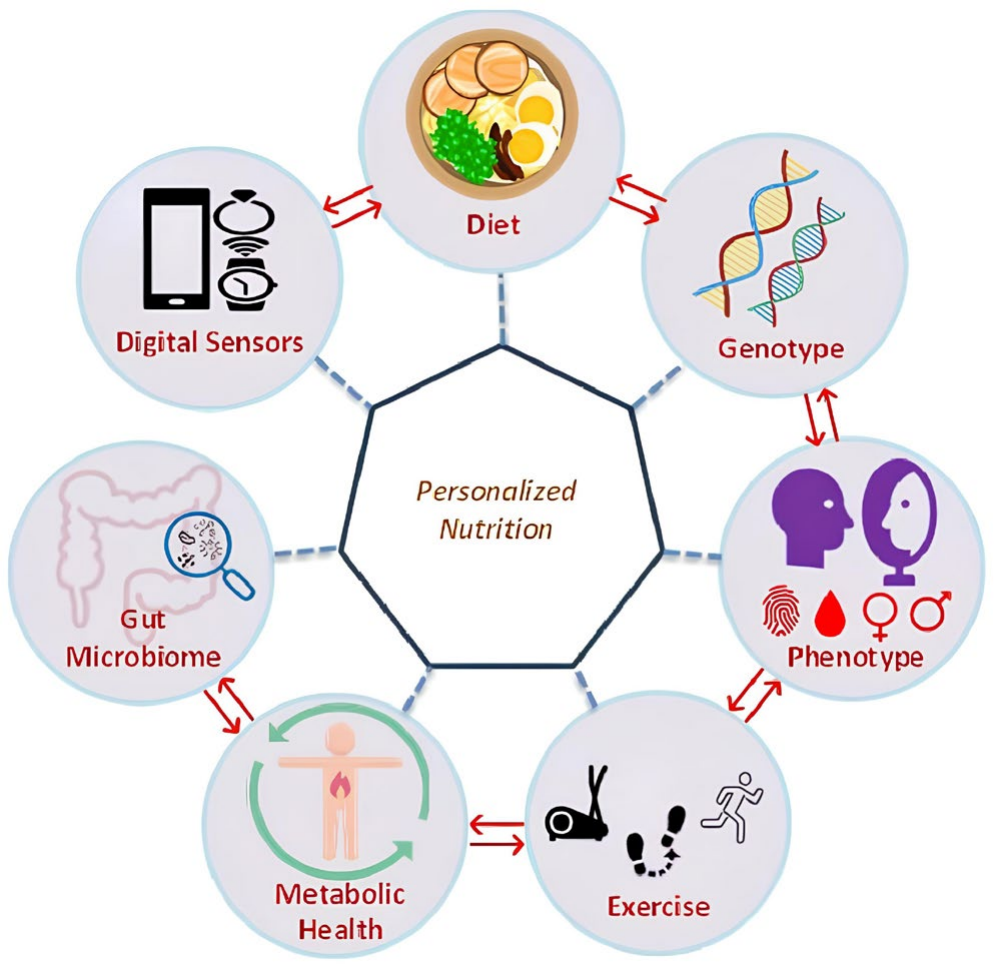
Components of personalized nutrition.

Metabolic well‐being can be improved with personalized prebiotic and probiotic therapy using microbiome profiling (Guizar‐Heredia et al. 2023). Novel digital health technologies, such as wearable sensors, AI apps, and CGMs, improve personalized nutrition by offering instantaneous feedback on the effect of food choices (Sempionatto et al. 2021; Shamanna et al. 2024). According to Ben‐Yacov and Rein (2022), the first tools enable people with obesity or diabetes to tailor their macronutrient content, meal timing, and food portions according to their own physiological response. Although concerns regarding data privacy, affordability, and clinical proof persist, additional indications and regulatory frameworks are essential to guarantee fair usage (Kohlmeier et al. 2016; Verma et al. 2018). Personalized nutrition can potentially revolutionize the prevention and management of chronic diseases, particularly with the emergence of precision medicine (Figure 1) (Moore 2020). Figure 1 shows the components of personalized nutrition.

### 2.1 Personalized Nutrition Based on Genetics

Personalized nutrition is a revolution that displaces blanket dietary advice with individualized meal plans based on metabolic, microbial, and genetic information (Bashiardes et al. 2018). Genetic variation has a significant influence on nutritional metabolism and affects the risk of obesity and diabetes (Drabsch and Holzapfel 2019). For example, de Toro‐Martín et al. (2017) established that SNPs in FTO and TCF7L2 are correlated with an increased risk of obesity and impaired glucose metabolism. As nutrigenomics science authorizes, carbohydrate sensitivity has a genetic origin, and low‐glycemic foodstuffs are better for some than for others (Zeisel 2020).

Personalized gene‐driven meals are superior to generic approaches for weight loss and glucose control (Matusheski et al. 2021). One study found that people who carried a mutation within PPARG gained additional benefit from consuming a Mediterranean diet that confined advanced amounts of monounsaturated fat (Ferguson et al. 2016). Correspondingly, to avoid metabolic disorders, patients with APOA2 polymorphisms must decrease saturated fat consumption (Lagoumintzis and Patrinos 2023). Depending on these results, personalized nutrition therapy for obesity and diabetes management can be achieved via nutrigenetic testing (Goni et al. 2016). The translation of genetic information into effective dietary plans continues to be plagued by several issues (Verma et al. 2018). Data protection and the risk of genetic determinism are two ethical issues that must be considered (Kohlmeier et al. 2016). Individualized nutrition plans can be augmented by merging genomic information with digital health technology despite these challenges (Moore 2020).

### 2.2 The Role of the Gut Microbiome in Personalized Nutrition

Nutrient uptake, inflammation, and metabolic health are considerably exaggerated by the gut flora (Kolodziejczyk et al. 2019). Certain bacterial species, as per a study by Guizar‐Heredia et al. (2023), comprising *A. muciniphila* , have been associated with improved insulin sensitivity, indicating that microbial diversity plays a role in obesity and diabetes pathogenesis. Leshem et al. (2020) established that augmented obesity and glucose intolerance are linked to dysbiosis, which is a disturbance in gut microorganisms. To facilitate microbial balance, personalized nutrition applies microbiome profiling to recommend prebiotic‐ and probiotic‐rich foods (Yeşilyurt et al. 2022). Individuals who are predisposed to diabetes can benefit from a high‐fiber diet because it enhances the production of short‐chain fatty acids (SCFAs) by the body (Christensen et al. 2018).

Zeevi et al. (2015) reported that nutritional advice based on microbiota enhanced postprandial glucose responses better than low‐carbohydrate recommendations. This evidence is reinforced by trials such as the PREDICT trials. New technologies, including microbiome analysis driven by AI, enable instantaneous dietary modification (Romero‐Tapiador et al. 2023). The high cost of microbiome testing prevents it from being widely adopted, and more research is required to ascertain its long‐term effectiveness (Torres and Tovar 2021). Palmnäs et al. (2020) stated that metabolic diseases can be treated with a microbiome‐targeted diet in the future, as metabolomics and metagenomics are still evolving.

### 2.3 Metabolic Response Monitoring and Digital Tools

Through real‐time metabolic feedback, wearable sensors and CGMs enable dynamic dietary modifications (Sempionatto et al. 2021). CGMs enable diabetics to optimize the timing and macronutrient composition of meals by showing the impact of different foods on blood glucose (Ben‐Yacov and Rein 2022). Individualized CGM‐based meals are more effective in reducing HbA1c levels than are standard treatments (Table 1) (Schembre et al. 2020). Table 1 shows the key components and tools for personalized nutrition: insights from current research.

**TABLE 1:** Key components and tools of personalized nutrition: insights from current research.

Aspect	Description	Tools used	Key insight	References
Genetics	Use of DNA variations to guide diet	Genotyping tests	Tailored nutrition plans based on genetic predisposition	Bashiardes et al. (2018)
Microbiome	Role of gut flora in dietary responses	Microbiome sequencing	Microbial diversity impacts nutrient absorption	Kolodziejczyk et al. (2019)
Metabolic response	Individual postprandial glucose variability	Continuous glucose monitors (CGMs)	Predicting glycemic response to foods	Zeevi et al. (2015)
Personalized apps	Mobile applications for tracking diet and metabolism	Mobile apps	User feedback loops for diet adjustments	Mortazavi and Gutierrez‐Osuna (2023)
Wearables	Real‐time health monitoring devices	Wearables (e.g., Fitbit)	Integrating physical activity with dietary intake	Sempionatto et al. (2021)
Precision nutrition	Customized diets based on metabolism	Biomarker analysis tools	Metabolic heterogeneity addressed through diets	Zeisel (2020)
Diet–microbiota interactions	Effects of diet on gut microbiota	Stool sample analysis	Personalized prebiotic and probiotic use	Torres and Tovar (2021)
Nutrigenomics	Gene–diet interactions	Genomic databases	Prevention strategies for chronic diseases	Ferguson et al. (2016)
Metabotyping	Phenotypic grouping for nutrition strategies	Blood biomarker profiling	Targeted dietary interventions	Palmnäs et al. (2020)
Postprandial responses	Glycemic and lipid response to foods	CGMs	Individualized meal planning	Guizar‐Heredia et al. (2023)
Nutritional AI	AI‐driven dietary recommendations	AI‐powered mobile apps	Predictive analytics for diet success	Romero‐Tapiador et al. (2023)
Digital biomarkers	Predictive models for meal timing	Wearable sensors	Enhancing diet timing precision	van den Brink et al. (2022)
Epigenetics	Nutritional effects on gene expression	DNA methylation analysis tools	Diet‐driven epigenetic modifications	Goni et al. (2016)
Consumer genomics	Rise of direct‐to‐consumer genetic testing	At‐home test kits	Democratization of personalized nutrition	Moore (2020)
Behavioral monitoring	Tracking user habits for dietary adaptation	Mobile/wearable integration	Behavior‐informed diet adjustment	Zahedani et al. (2023)
Digital twins	Predictive glycemic modeling for individuals	Digital twin platforms	Improved diabetes remission via nutrition	Shamanna et al. (2024)
Wearable validation	Accuracy of nutrition‐tracking wearables	Controlled trials	Validating intake measurement devices	Dimitratos et al. (2020)
Gut microbiome enterotypes	Categorizing users by gut types for diets	Metagenomic analysis	Precision probiotics for obesity management	Christensen et al. (2018)
Future of personalized nutrition	Integrating IoT and AI for diet plans	Smart devices and cloud analytics	Tailored dietary solutions via smart systems	Priyadharshini et al. (2025)
Challenges in implementation	Ethical, scientific, and technical challenges	Multimodal data collection platforms	Balancing innovation with validation	Verma et al. (2018)

To develop individualized meal plans, AI platforms and mobile applications integrate information on glucose, microbiota, and genetics (Mortazavi and Gutierrez‐Osuna 2023).

Zahedani et al. (2023) referred to ZOE's personalized nutrition program as an example that employs CGM data and microbiota analysis to predict glycemic responses. Biosensors and smartwatches track calorie intake, sleep, and exercise, which enhance compliance (Dimitratos et al. 2020). Digital technologies hold promise, but there are barriers to their use and availability (Liao and Schembre 2018). Additionally, the triglyceride–glucose (TyG) index and its derivatives (e.g., TyG–WHtR) have emerged as independent predictors of all‐cause and cardiovascular mortality in hypertensive patients, suggesting their application in digital risk assessment models (Li et al. 2025). Such markers, along with CGM and AI systems, can be used to advance personalized risk stratification for metabolic disease care.

Through the simulation of metabolic reactions, emerging technologies such as digital twins can further enhance individualized nutrition in the future (Shamanna et al. 2024). A new game‐changing model for managing diabetes and obesity is personalized nutrition, which is powered by genetics, microbiome science, and digital health. This paradigm moves attention away from generic diets and toward targeted, individualized interventions based on the biology of each individual. To achieve full potential, problems concerning price, data privacy, and clinical evidence must be addressed.

## 3 Genetic and Epigenetic Influences on Nutrition

Obesity and diabetes can be more easily understood by reviewing the intricate dynamics of diet, epigenetics, and inheritance. Boland et al. (2019) and Mansour et al. (2024) reported that genetic variations, such as single nucleotide polymorphisms (SNPs), can affect metabolism, appetite regulation, and fat accumulation by altering the way individuals react to different nutrients. For instance, many indications are associated with the risk of obesity with gene polymorphisms, such as FTO and MC4R, predominantly when coupled with high‐calorie diets (Heianza and Qi 2017; Gkouskou et al. 2024). Diet can affect genetic risk, as shown by nutrigenomics (the study of nutrient impact on gene expression), which demonstrates that personalized nutrition can decrease the severity of disease (Marcum 2020).

Furthermore, recent advancements in genomics and bioinformatics have made it possible to discover how diverse nutrients affect gene expression. This has opened the door for precision nutrition methods that aim to minimize the load of metabolic diseases (Vincenti et al. 2024; Ndimele et al. 2017). With respect to modulation of the impact of early‐life and lifetime dietary acquaintance, epigenetic modifications—heritable alterations in gene expression that are not DNA sequence changes—are as essential as genetic susceptibility (Burgio et al. 2015). Dietary circumstances can influence non‐coding RNAs, DNA methylation, and histone modifications, thus influencing metabolic well‐being from generation to generation (Franzago et al. 2019; Mishra et al. 2022). If the mother does not eat enough while pregnant, she might have genes that are “hardwired” to create children who become diabetic, obese, and predisposed to other metabolic disorders (Miraghajani et al. 2017). Folate and polyphenols are two recently discovered nutrients that can reverse pathogenic epigenetic markers and have potential therapeutic applications (Elsayed and Saleh 2024; Zeinalian et al. 2022). Understanding the dual actions of genetic susceptibility and nutritional epigenetics is crucial for appropriate intervention in the multifactorial etiology of diabetes and obesity.

### 3.1 Nutrigenomics and Obesity/Diabetes Risk

Increasing evidence suggests that diet and genetic predispositions contribute significantly to the development of metabolic syndrome and T2D. Nutrigenomics, according to Boland et al. (2019), is all about how various individuals' genes influence their bodies' responses to nutrition and how this impacts their metabolic health. Others are more prone to developing diabetes or obesity even when eating the same diet, and scientists have begun to grasp this by examining gene expression differences due to specific dietary factors. An example is the link between high‐fat diets and specific genetic variations, such as those in the FTO and TCF7L2 genes, which make a person more likely to develop diabetes (Marcum 2020). Increasing evidence is revealing that environmental and genetic interactions result in obesity and diabetes, not vice versa (Burgio et al. 2015).

Genetically influenced nutritional impacts controlled by gene mutations and SNPs have been the focus of increasing research owing to advances in nutrigenomic technologies. The way in which nutrients are digested and utilized by the organism is influenced by genetic differences; these differences influence the emergence and progression of metabolic disorders, specifically (Zeinalian et al. 2022).

Recent studies have demonstrated that epigenetic alterations, including modifications of DNA methylation and histone changes, control the long‐term influence of early food exposure on the risk of disease. An example is the association between metabolic dysregulation and epigenetic programming in children, which can arise when mothers fail to eat nutritious food throughout pregnancy (Franzago et al. 2019). This aids in designating the vulnerable time of pregnancy, where nutrition–gene interactions determine the trajectory of future health. In particular, there are food remedies with the motivation of plummeting the risk of illness via epigenetic indicators. Studies have shown that certain nutrients, such as folate, choline, and polyphenols, can moderate the patterns of DNA methylation complexes in the development of obesity and diabetes (Mishra et al. 2022). These explanations support the argument that personalized dietary advice can benefit from the amalgamation of epigenetic data. Currently, genetic, epigenetic, and microbiome data are obtained using high‐throughput technologies such as genome‐wide association studies (GWAS), DNA methylation profiling, and 16S rRNA gene sequencing (Demirkan et al. 2024). These platforms enable the detection of polymorphisms (e.g., FTO and TCF7L2), methylation signatures, and gut microbial profiles associated with metabolic dysfunction. Personalized nutrition sites integrate omics information with evidence‐based approaches that translate biological profiles into dietary recommendations (Ramos‐Lopez et al. 2022). For example, nutrigenetic tests translate SNPs to personalized macronutrient recommendations, whereas microbiome‐assisted applications screen taxa such as *A. muciniphila* and *Faecalibacterium prausnitzii* to forecast glycemic effects (Singar et al. 2024). The major biomarkers of diabetes and obesity are fasting glucose, HbA1c, the triglyceride–glucose (TyG) index, HOMA‐IR, pro‐inflammatory cytokines (e.g., IL‐6 and TNF‐α), and microbiota‐derived SCFAs (Anachad et al. 2023). In addition to genetic and epigenetic elements, bile acids (BAs) are being increasingly identified as regulators of obesity. Mendelian randomization analyses suggest a potential inverse causal association of glycolithocholate (GLCA) trunk fat percentage, indicating a role for BA metabolism in obesity control (Huang et al. 2024). These observations broaden our understanding of metabolic targets for precision nutrition interventions.

More strongly, diet has been associated with a greater risk of T2D, leading to large cohort studies. The prospective dietary lifestyle to nullify genetic predispositions was emphasized in an exploration by Jia et al. (2021), where high fruit consumption abridged the genetic risk for T2D. A new area of study referred to as “precision nutrition” is using genetic data to tailor treatments that can avert or postpone metabolic disease. According to Gkouskou et al. (2024), this strategy goes beyond simple food recommendations by providing individualized programs that render individual genetic profiles to each person. With the global rise in noncommunicable diseases, such innovation is more crucial than ever.

The translation of nutrigenomic results into clinical application remains plagued by challenges, even with encouraging progress. There must be heterogeneity in cohort studies and methodological standardization to address study design heterogeneity, demographic heterogeneity, and uneven replication of findings (Mondal and Panda 2021). Issues regarding genetic privacy must be resolved as precision nutrition has become increasingly prevalent in medicine. Nonetheless, a revolutionary advancement in the management of diabetes and obesity is the acknowledgment of nutrigenomic effects. Mansour et al. (2024) advise that scientists continue to refine gene‐diet models and that nutritional interventions aimed at specific diseases should be tested and confirmed through extensive clinical trials. Tailor‐made intervention strategies may be elevated to the next level by integrating omics information with machine learning (ML) and wearable electronics (Ndimele et al. 2017).

### 3.2 Gene–Diet Interactions

Individuals have various vulnerabilities to illness and varying reactions to diet therapies, which are largely a result of gene–diet interactions. Heianza and Qi (2017) state that these interactions are conditions in which an individual's genetics determine how dietary components influence health outcomes. For example, individuals carrying certain FTO variants are likely to become obese if they consume high amounts of calories, but the risk can be minimized if they maintain healthy eating behaviors (Zhuang et al. 2021).

Research on metabolic issues has indicated that genetics can elevate or reduce the influence of food constituents on disease risk. Of particular note regarding diabetes therapy, Westerman et al. (2021) employed the UK Biobank data to illustrate how certain dietary–gene pairings influence hemoglobin A1c. These results reinforce the potential for dramatic improvement in disease outcomes with customized diet plans based on genetic information. Furthermore, food influences on obesity and the growth of diabetes are regulated by differences in genes regulating inflammatory processes, glucose metabolism, and lipid metabolism (Crovesy and Rosado 2019). The risk of diabetes is controlled by triglyceride levels, which are regulated by polymorphisms in the APOA5 gene and ingestion of dietary fats (Ortega et al. 2017). To predict the risk of obesity with better precision, investigators have progressively turned to ML methods to examine intricate gene–diet interactions (Lee et al. 2022). Using these approaches, investigators can obtain vast amounts of genetic and nutritional data so that they are capable of detecting slight interface effects that have not been observed with more conventional approaches. Investigations suggest that exposure to the environment and genetics could affect interactions between diet and gene populations. Sekar et al. (2023) reported that research into interactions between genes and food in Southeast Asian populations has identified population‐specific genetic variations that affect the risk of diabetes compared to Western populations. These results highlight the importance of learning about numerous ethnic populations in an effort to develop personalized nutritional recommendations that are applicable globally (Table 2). Table 2 shows the genetic and epigenetic influences on nutrition: nutrigenomics and gene–diet interactions in obesity and diabetes risk.

**TABLE 2:** Genetic and epigenetic influences on nutrition: nutrigenomics and gene–diet interactions in obesity and diabetes risk.

Topic	Key findings	Nutritional implications	Future directions	References
Nutrigenomics and obesity	Genetic variants impact obesity risk via nutrient metabolism pathways	Personalized diets can reduce obesity risk	Integrating genetic testing into routine care	Boland et al. (2019)
Nutrigenomics in athletes	Genetics may dictate optimal macronutrient ratios for performance	Tailored diets enhance athletic outcomes	Further exploration of sports‐specific nutrigenomics	Vincenti et al. (2024)
Genetics of obesity	Epigenetic changes can predispose individuals to obesity	Early nutritional interventions can reverse epigenetic marks	Preventive nutrition strategies	Burgio et al. (2015)
Gestational diabetes & epigenetics	Maternal diet influences fetal epigenome and diabetes risk	Nutritional counseling in pregnancy is vital	Long‐term monitoring of offspring	Franzago et al. (2019)
Mineral deficiencies & nutrigenomics	Electrolyte imbalances interact with genetic predispositions	Correction of deficiencies may lower metabolic risks	Mineral‐genetics studies	Mondal and Panda (2021)
Personalized nutrition in diabetes	Genetic profiles can guide dietary management	Precision diets can improve glycemic control	Wider application of nutrigenomics	Zeinalian et al. (2022)
Nutrigenetics in healthcare	Personalized nutrition can transform healthcare systems	Disease prevention through diet	Policy implementation	Marcum (2020)
Microbiota and obesity genetics	Gut microbiota interplay with gene–nutrient interactions	Microbiota modulation for obesity treatment	Probiotic‐genetic interventions	Mansour et al. (2024)
Nutritional genomics review	Different genes respond uniquely to the same nutrient	Need for genotype‐specific recommendations	Expansion of databases	Elsayed and Saleh (2024)
Gene‐nutrition‐health interface	Nutrigenomics links diet, gene expression, and disease	Holistic nutritional approaches	Interdisciplinary research	Mishra et al. (2022)
Prenatal nutrition exposure	Intrauterine diet programs future obesity risk	Targeting maternal nutrition	Preventive public health policies	Miraghajani et al. (2017)
OMICS platforms in metabolism	Integrated OMICS improve precision nutrition	Data‐driven dietary interventions	Technological integration in clinics	Ndimele et al. (2017)
Childhood obesity & precision nutrition	Early‐life genetics influence obesity risk	Personalized interventions in childhood	Lifelong nutritional monitoring	Wu et al. (2020)
Obesity genomics and cardiometabolic risk	Metabolomic profiles reveal obesity–genetics connections	Metabolite‐targeted diets	Biomarker discovery	Regan and Shah (2020)
Genetics, nutrition, and disease prevention	Genetic understanding can predict disease risk	Preventive nutrition strategies	Genomic literacy in nutritionists	Agrawal et al. (2024)
Nutrigenomics prospects	Nutrigenomics can redefine nutritional sciences	Emerging field with vast applications	Global nutrigenomic initiatives	Bahinipati et al. (2021)
Nutrigenomics in public health	Mass customization of diets based on genetics is possible	Public health nutrition programs	Feasibility studies	Reddy et al. (2018)
Personalized obesity prevention	Genomic insights enable obesity prevention	Early risk identification	Genome‐guided dietary planning	Gkouskou et al. (2024)
Gene‐diet interaction in obesity	Specific gene variants interact with diets to affect obesity	Tailored interventions based on risk alleles	Longitudinal studies needed	Heianza and Qi (2017)
Diet quality and genetic predisposition	Poor diet amplifies genetic risk for diabetes	High‐quality diets mitigate genetic risks	Gene–environment modification studies	Zhuang et al. (2021)

Diet patterns, rather than individual nutrients, are the targets of genetic risk research. Adherence to a healthy dietary pattern lowers the risk of obesity regardless of genetic predisposition, as demonstrated by Nettleton et al. (2015). This supports the contention that promoting healthy eating habits overall remains important, even when personalization is taken into account. In addition, the effectiveness of public health nutrition interventions can be influenced by gene–environment interactions. Recent experimental research has shown that microcystin‐LR (MC‐LR), a cyanotoxin, accelerates liver lipid metabolic disorders in obese mice through the PI3K/AKT/mTOR/SREBP1 pathway (Chu et al. 2022). The findings emphasize the interaction of environmental dietary toxins and metabolic gene regulation as central problems in personalized nutrition design.

For instance, universal dietary interventions that seek to reduce sugar intake may not be as effective for individuals who are genetically inclined to eat more sugar (Haslam et al. 2018). Therefore, to enhance the effectiveness of interventions, public health recommendations can be individualized based on genetic risk profiles. Encouraging results have been found, but a large challenge is the inconsistency across studies investigating gene–diet interactions. To approve deductions and create stable gene‐diet connections with applicability to real life, Dietrich et al. (2019) underscored the consequences of large‐scale, high‐quality studies. Personalized nutrition is achievable because of the enhanced understanding of gene‐diet connections, as well as the creation of new opportunities for diabetes and obesity treatment. Based on the latest examination (Vincenti et al. 2024; Elsayed and Saleh 2024), the successful determination and control of metabolic disease will most positively comprise the blending of genomic information with the continuous assessment of nutrition utilizing digital health technologies.

## 4 Real‐Time Data and Glucose Monitoring

Diabetes and obesity control have been greatly improved by the real‐time data collected through CGM. Glucose is measured minute by minute using CGMs, and people can see the impact of food, exercise, and medications in real time (Alfadhli et al. 2016; Hegedus et al. 2021). Patients can improve, take care of their diabetes themselves, and stick to their new way of life after being provided with constant feedback about their separate glycemic patterns. Patients with insulin‐treated diabetes had better quality glucose levels and more severe acute metabolic problems when they used real‐time CGMs, as described by Karter et al. (2021). In addition, CGM technology has been critical to obesity examination in revealing the connection between glucose variability and health risks linked to weight (Brummer et al. 2024). The course of T2D development from obesity can be slowed by incorporating CGMs into the standard medical practice. This makes early intrusion possible, which consequently allows for diet alteration and behavioral modification. The educational function of the CGM is also extremely important. Examinations have shown that access to real‐time glucose data can help people make healthier lifestyle and food choices. It is also used as an instrument to directly alter behavior directly (Ehrhardt and Al Zaghal 2019; Engler et al. 2022).

For instance, Alfian et al. (2018) highlighted how intelligent healthcare systems increase diabetes patients' self‐observational competences and condition monitoring by processing in‐the‐moment information from wearables. Additionally, studies have shown that CGM‐based treatments are helpful for patients who are not on insulin. This means that obese people can be helped by initial exposure to real‐time glucose response to avoid metabolic drop (Moon et al. 2023). In general, connecting real behavior with metabolic reactions and real‐time glucose monitoring is a paradigm shift in precision medicine.

### 4.1 Role of CGMs

By permitting them to track real‐time blood glucose changes, CGMs have been developed for diabetes management (Karter et al. 2021). They concentrate on the trends of hyperglycemia or hypoglycemia, while glucose is continuously measured day and night using this technology. By presenting the immediate effect of changes in food, exercise, stress, and medicine, this real‐time response has an important advantage over conventional fingerstick testing in diabetic patients (Engler et al. 2022).

Expanding the scope of glucose‐responsive innovation, a novel therapeutic gel has shown promise in healing diabetic subcutaneous abscesses through the synergistic action of photodynamic therapy, oxygen release, and pain killing induced by elevated glucose levels (Huang, An, et al. 2025; Huang, Huhulea, et al. 2025). These intelligent materials illustrate how metabolic signals can directly guide personalized care as an add‐on to digital feedback.

Consequently, CGMs are not only devices for more than analysis but can also be used as behavioral interferences to encourage people to uphold a better lifestyle. CGMs have been used extensively in obesity studies. The correlation between deranged glucose dynamics and weight management was explained by Hegedus et al. (2021), who found that even among obese non‐diabetic persons, real‐time glucose monitoring can detect personalized glycemic patterns. Obesity predisposes individuals to the development of T2D, and subtle disturbances in glucose control are often present before clinical diagnosis; therefore, these results are predominantly significant. Preventive treatment can be allowed through the use of CGMs by noticing the dysregulation of metabolism early on, prior to instigating irreparable harm. Furthermore, CGMs are used as educational tools, allowing patients to comprehend the impact of specific meals on their blood glucose levels (Alfadhli et al. 2016). For instance, CGM‐supported education allows individuals with gestational diabetes to augment their control of glucose and, perhaps, decrease the risk of adverse pregnancy outcomes. In addition, by illustrating the physiological impact of food selection graphically, CGMs may promote swift behavior changes in individuals with T2D and prediabetes (Ehrhardt and Al Zaghal 2020).

Both the precision and user experience of CGMs have been significantly enhanced by technological innovations. One of the devices that minimizes the user burden is a factory‐calibrated CGM (Tripyla et al. 2020). Teenagers, adults, and non‐insulin users have found such novelty acceptable to studies (Moon et al. 2023; Liao and Schembre 2018). As they become more reachable, CGMs will be in a better position to influence public health interventions for opposing growing rates of obesity and diabetes. One crucial integral of diabetes treatment is behavior modification, and CGMs deliver a novel process to encourage behavioral change through feedback‐based data (Ehrhardt and Al Zaghal 2019). Continuously guided mats assist individuals in developing habits and adhering to good lifestyle decisions over time, as long as they have real‐life significance for their exercise and food choices. Better than broad dietary advice would be to present to a patient how a meal that is high in carbohydrates causes a rapid rise in blood sugar. Greater compensation has been observed when CGMs are part of lifestyle interference programs. A pilot study by Taylor et al. (2019) proved that CGMs enhanced glycemic control in patients with T2D more than lifestyle counseling. Participants could personalize their responses based on real‐time information, resulting in a more adaptive and individualized approach to diabetes management. Not only will CGMs be useful for modifying behavior, they will also reduce the likelihood of long‐term diabetic complications by reducing glucose variability as a risk factor for cardiovascular complications (Jamiołkowska et al. 2016). These patients can minimize the risk of developing diabetic cardiovascular complications by minimizing their blood glucose excursions and, in return, decreasing their risk for oxidative stress and endothelial dysfunction. Other applications of CGMs beyond glucose control have been explored. New avenues for the examination of eating behavior in obesity studies have become available since Brummer et al. (2024) highlighted the potential for CGMs to automatically register eating episodes. Key information that is often lost using conventional food diaries, this device is able to offer quantitative and qualitative information regarding eating patterns, meal frequency, and glucose responses. Finally, CGMs can become an ordinary instrument in diabetes treatment and preventive health care, given that their costs continue to decline as insurance coverage increases. There is hope that CGMs have the potential to reduce the social burden of obesity and T2D by monitoring those at risk and enabling them to make early, individualized interventions (Porter et al. 2022).

### 4.2 AI‐Driven Meal Planning and Feedback Loops

New innovative meal planning software has been introduced by AI, which provides personalized dietary recommendations based on health information, lifestyle behavior, and metabolic requirements. A significant component of AI‐based meal planning for obesity and diabetes is the generation of personalized nutritional therapy that assists in blood glucose level regulation and the maintenance of weight management (Mehrotra and Mehrotra 2024). For example, a new research study by Xiong et al. (2024) developed a personalized light‐based prediction model for the prediction of postprandial glycemic response (PPGR) of type 1 diabetes patients with significantly better predictive performance (*R* = 0.63) than traditional carbohydrate‐counting models (*R* = 0.14) and traditional insulin infusion techniques (*R* = 0.43) (Xiong et al. 2024).

In another study, Bhadouria and Ahirwar (2024) employed a Random Forest classifier in the Nutrition Diet Expert System (NDES) to offer personalized dietary recommendations to diabetic patients with 96.48% accuracy, 0.98 precision, 0.96 recall, and an F1‐score of 0.97, indicating the potential of AI to improve glycemic control and patient outcomes (Bhadouria and Ahirwar 2024).

Unlike the general nature of the traditional diet plans, AI models can analyze real‐time CGM data and modify recommendations to maximize glycemic control (Joachim et al. 2022). The ability of AI to scan enormous amounts of individual health information, including real‐time glucose readings, activity levels, and sleep patterns, to provide personalized nutritional advice is a significant advantage of AI for meal planning. Considering the advantages of AI‐based nutrition apps for diet and health improvement, Prasad et al. (2025) highlighted the manner in which such apps enable individuals with diabetes to manage their blood sugar levels better and more effectively by adapting recommendations according to user‐specific glycemic responses. Several clinical and real‐world studies have demonstrated the efficacy of digital nutritional interventions in improving metabolic outcomes. For instance, CGM‐based dietary feedback has been shown to significantly lower HbA1c levels in T2D patients compared to standard self‐monitoring (Shamanna et al. 2024; De Luca et al. 2023). Similarly, mobile health apps combined with remote counseling have led to notable reductions in body weight and improvements in insulin sensitivity (Kitazawa et al. 2024), supporting their integration into broader dietary risk mitigation strategies.

Obese individuals, whose metabolic responses to food vary widely, stand to gain significantly from such tailoring (Nehete 2023). In addition, feedback loops brought about by AI systems allow for ongoing learning and real‐time eating plan refinement (Nehete 2023) for consumers. One of the novel mobile app prototypes, HealthyBaby by Al‐Massoudi et al. (2022), uses a logistic regression classifier and deep learning regression combined to provide personalized pregnancy diet recommendations with improved performance over conventional static methods. The integrated model accounted for user‐specific dietary restrictions and nutritional needs, indicating the growing applications of AI in managing maternal nutrition (Al‐Massoudi et al. 2022).

Glucose excursions can be evaluated, and real‐time feedback and meal adjustment recommendations can be provided by the system following every meal. Owing to this continuous feedback loop, individuals are likely to eat healthily and are able to make adjustments prior to any opposing metabolic consequences. Once AI enables patients to accomplish their own diabetes management, active self‐management is the outcome of passive compliance (Huang, An, et al. 2025; Huang, Huhulea, et al. 2025). Scientists have also established that AI‐based predictive models can prevent obese people from developing diabetes. According to Huang, An, et al. (2025) and Huang, Huhulea, et al. (2025), AI may use biomarker data in real time to forecast the risk of obesity‐related situations such as diabetes and propose diet modifications to delay them. AI‐supported dietary interventions in the early stages can delay or prevent the development of obesity in T2D by improving metabolic flexibility and maintaining glucose homeostasis (Nehete 2023).

Further, AI‐powered platforms have engaged in behavior modification techniques, such as gamification, nudges, and motivational feedback, to sustain individuals on the diet track. Such plans promote a user interface with diabetes self‐management applications, which result in lasting lifestyle changes (Priesterroth et al. 2019). People with diabetes and obesity can benefit significantly from intelligence's capability to keep them on their toes about their long‐term work towards altering their behavior (Hemanth et al. 2025). AI‐powered meal plans can be as intricate as desired, more than just calorie and macronutrient distribution. Diet plans that exploit hormonal equilibrium, fullness, and glycemic burden are recommended by AI systems (Hemanth et al. 2025), which are critical parameters in diabetes and obesity management. AI can plan diets that provide blood sugar and enhance metabolic health by considering the complex interactions between nutrients and metabolic pathways.

Polyphenols from *Myrica rubra* pomace have also exhibited hypoglycemic and gut microbiota‐modulating effects in T2D mouse models by modulating the PI3K and AMPK signaling pathways and enhancing GLUT‐4 and IRS‐1 expression (Chang et al. 2025). Such bioactive compounds can be utilized as key components in algorithm‐based meal planning in precision nutrition systems.

With the advent of mobile apps, wearable devices, and CGM integration, AI‐based diet management is becoming more common. In their impression of AI's role in diabetes management, Sarma and Devi (2025) emphasized how technology is streamlining patient‐doctor communication, leading the way for remote monitoring and real‐time decision support. To inspire quicker and healthier eating habits, they allow users to receive meal suggestions based on their current glucose patterns. People may more readily heed dietary advice without feeling deprived as a result of AI's adjustability, respecting cultural, territorial, and personal eating habits. For improving insulin sensitivity and lowering the threat of obesity, chrononutrition (diet in accordance with natural circadian cycles) plays an important part, and AI's potential was examined by Bajaj and Lata (2024) in this application. Both the quantity and timing of individuals' consumption can be further optimized in such a manner so as to stimulate the best overall metabolic health (Table 3). Table 3 shows the role of CGM‐ and AI‐driven nutrition in diabetes and obesity management.

**TABLE 3:** The role of continuous glucose monitoring (CGM) and AI‐driven nutrition in diabetes and obesity management.

Technology	Application	Key findings	Population studied	References
CGM	Educational tool for gestational diabetes	Improved glycemic control and patient understanding of food impacts	Pregnant women with GDM	Alfadhli et al. (2016)
CGM	Obesity research and dietary behavior tracking	Identified postprandial glucose variability linked to obesity	Adults with obesity	Hegedus et al. (2021)
CGM + AI	Real‐time eating event detection	Automated meal logging reduced user burden and improved accuracy	General population	Brummer et al. (2024)
AI/ML algorithms	Obesity and heart disease risk prediction	Enhanced real‐time risk stratification using glucose patterns	High‐risk cardiometabolic patients	Devarapu et al. (2019)
Bluetooth‐enabled CGM	Personalized diabetes monitoring system	Reduced hypoglycemia episodes through real‐time alerts	T2D patients	Alfian et al. (2018)
CGM	Behavioral intervention for T2D	Increased physical activity and dietary adherence	Adults with T2D	Engler et al. (2022)
Digital tracker + CGM	Glucose regulation improvement	Significant HbA1c reduction in healthy and T2D users	Healthy adults & T2D patients	Zahedani et al. (2023)
CGM	Prediabetes behavior modification	Enhanced patient engagement in lifestyle changes	Prediabetic adults	Ehrhardt and Al Zaghal (2019)
CGM	Glycemic control in insulin‐treated diabetes	Reduced severe hypoglycemia events	T1D and insulin‐dependent T2D	Karter et al. (2021)
CGM	Pilot self‐care support for T2D	Improved meal timing and portion control	T2D patients	Porter et al. (2022)
AI‐driven meal planning	Nudge‐based diabetes self‐management	Increased adherence to Mediterranean diet	T2D patients	Joachim et al. (2022)
AI nutrition assistant	Personalized diet recommendations	Optimized macronutrient distribution based on glucose trends	T2D and obese patients	Nehete (2023)
AI + CGM integration	Dynamic meal planning	Reduced postprandial glucose spikes by 27%	T2D patients	Mehrotra and Mehrotra (2024)
AI analytics	Obesity risk prediction	Early identification of metabolic deterioration patterns	High‐BMI adults	Huang, An, et al. (2025) and Huang, Huhulea, et al. (2025)
Gamification + CGM	Diabetes self‐management apps	Improved long‐term engagement through reward systems	T1D and T2D patients	Priesterroth et al. (2019)
Social incentive apps	Lifestyle modification in diabetes	15% greater weight loss vs. control group	Uncontrolled T2D	Patel et al. (2021)
AI‐powered WHO guidelines	Interactive nutrition education	Improved dietary knowledge retention	General and diabetic populations	Ojo et al. (2025)
Machine learning	Precision meal plans for PCOS	Balanced insulin and androgen levels through customized diets	PCOS patients	Hemanth et al. (2025)
AI‐driven chrononutrition	Circadian‐based meal timing	Reduced nocturnal glucose variability	Shift workers with T2D	Bajaj and Lata (2024)
CGM + wearable sensors	Metabolite tracking for wellness	Real‐time micronutrient adjustment based on glucose‐metabolite correlations	Health‐conscious users	Banka et al. (2025)

Although AI meal planning promises much, there are challenges ahead, including concerns regarding data privacy and algorithm transparency. Mackenzie et al. (2024) emphasized the need to address any biases in AI models and to ensure ethical use. Despite some challenges, the assistance of AI in enhancing nutrition approaches for diabetes and obesity control is substantial, pointing towards a good future for precision health interference. Finally, diabetes and obesity care have experienced a paradigm shift with the advent of AI‐driven feedback loops and meal planning. They allow people to play an active and educated role in their own healthcare process via hyper‐personalized real‐time nutritional guidance, enhancing glycemic control and weight loss (Kassem et al. 2025).

### 4.3 Comparison of Digital Tools for Dietary Monitoring and Exposure Management

The recent past has witnessed a speeding up in the adoption of digital technologies within diet assessment and health surveillance, particularly in managing the consumption of harmful food substances and improving metabolic outcomes. Digital health technologies, such as CGMs, mobile applications, wearable physical activity monitors, and AI systems, offer promising tools for personalized nutrition counseling and enabling behavior‐change interventions (Table 4) (Edelman et al. 2018; Pang et al. 2018; Dhar et al. 2023; Fanelli et al. 2023; Vegesna 2024). These online tools have the potential to reduce the intake of detrimental dietary components such as heavy metals by encouraging healthy food choices, tracking packaged food intake, and offering personal alerts or education. Table 4 summarizes the major tools, their purposes, target populations, primary features, and current limitations.

**TABLE 4:** Comparison of digital tools for dietary monitoring and exposure management.

Technology/tool	Function	Target users	Key features	Limitations	References
CGMs	Tracks glucose response to diet	T1DM, T2DM	Real‐time alerts, dietary trend detection	Not specific to heavy metals; may cause skin irritation or discomfort	Edelman et al. (2018)
Mobile health apps	Logs food intake and risks	General public, obesity, T2DM	Barcode scanning, alerts on additives/metals	Limited database coverage; user adherence often declines over time	Pang et al. (2018)
Wearable trackers	Monitors physical activity & calorie intake	Obesity, fitness‐focused individuals	Real‐time health data, sync with diet apps	Doesn't measure contaminants; overestimation or underestimation of energy expenditure	Dhar et al. (2023)
AI‐based nutrition apps	Generates personalized diet plans	Obesity, metabolic syndrome	AI‐driven recommendations, food risk evaluation	Varying accuracy; recommendations may not be culturally or regionally tailored	Vegesna (2024)
Telenutrition platforms	Offers remote dietary counseling	At‐risk, chronically ill populations	Expert guidance, behavior modification	Requires digital access and literacy; limited in physical assessment capabilities	Fanelli et al. (2023)

## 5 Behavioral Change Through Technology

To decrease the severity of diabetes and obesity, digital adherence policies, gamification, and nudges can be employed to stimulate healthy lifestyles. Including game‐like elements such as rewards, challenges, and social competition in gamification policies has been found to meaningfully improve engagement and support good behavior. Gamification‐based DM applications employ behavior‐change techniques to empower patients to adhere to treatment (Priesterroth et al. 2019). Correspondingly, for people with uncontrolled diabetes, a randomized clinical trial combining gamification with social incentives showed that such people accomplished significant lifestyle alterations. Nudges, or small design vicissitudes that quietly encourage healthier choices, have also been shown to be essential in this field. Nudges, comprising social comparisons and goal reminders, can influence health behaviors without constraining separate choices (Kwan et al. 2020). This makes them an operative tool for the management and prevention of diabetes.

Digital channels modified by incorporating AI and ML boost behavioral change policies significantly. Banka et al. (2025) recognized the significance of AI‐driven customization in the distribution of tailored, responsive diet and physical activity treatments, as their investigation established that interventions improved compliance and health outcomes. Experts have recognized that gamification, if added to incentives or simulations, significantly improves the chance that people will exercise and adhere to medication according to instructions (Agrawal et al. 2024; Agarwal et al. 2021). Current digital health technologies often lack an in‐depth behavioral theory, as stated by Klonoff (2019), although such a theory is significant for sustaining change over the long term. To exploit the efficiency of digital interventions in diabetes and obesity deterrence programs, they should ideally be informed by behavioral science, clinically established (Gomis‐Pastor et al. 2024), and centered on user preferences (Berger and Jung 2024).

### 5.1 Gamification, Nudges, and Adherence

Over the past few years, gamification has emerged as an effective tool for engaging individuals with diabetes and obesity to change their behavior. As Priesterroth et al. (2019) report, patients will be more inclined to participate and adhere to treatment programs if self‐management apps feature game elements, such as points, levels, and rewards. Users of the diabetes app indicated greater levels of app use, self‐efficacy, and glycemic control following gamified therapy participation. By incorporating interactive and personalized elements, Orte et al. (2023) developed a gamified mobile health framework to promote healthy eating behaviors. Diabetes control has also been gained from the inclusion of nudges, which are small design cues that nudge individuals towards improved choices without encroaching upon their autonomy (Orte et al. 2023).

Personalized reminders, social comparisons, and default options significantly boosted health behavior among people managing diabetes, as established by Kwan et al.'s (2020) wide‐ranging review of nudge strategies. Digital health interventions that include behavioral nudges, as noted by Shah and Adusumalli (2020), can significantly increase the adoption and continuous use of health interventions, which are crucial for the prevention of diabetes and obesity. The impact of gamification interventions aimed at specific behaviors has been established through large‐scale clinical studies. Overweight and obese adults in the United States experienced a significant increase in their physical activity levels after they were randomly assigned to participate in STEP UP, as conducted by Patel et al. (2019). Similarly, the MOVE‐MORE program showed the potential for a mix of digital and environmental interventions by prompting office workers to take walking breaks through web‐based gamification and physical nudges (Mamede et al. 2021).

A new realization is that customized gamification is the most effective. As Banka et al. (2025) point out, one of the ways to enhance accuracy in wellness methods is through the application of AI and ML to customize gamification according to each individual's habits, preferences, and health goals. Individuals with diabetes and obesity are more likely to adhere to the lifestyle modifications required, such as consuming healthier foods and exercising more, if they have a plan specifically tailored to their requirements. However, it is also important to add behavioral theory to digital platforms to achieve effective behavioral changes. Most digital health interventions fail, as Klonoff (2019) opines, because they fail to integrate the three pillars of behavior change: goal setting, self‐monitoring, and reinforcement. Adding these theories to gamified platforms increases participation and encourages users to keep up their behavior in the long term, which is fundamental in preventing chronic diseases. Further gains were found when gamification was combined with financial incentives. Agarwal et al. (2021) found that gamified exercise programs with financial incentives support a significantly greater level of adherence. This indicates that gamified experiences can add to intrinsic motivation, particularly in populations that are at risk for obesity and diabetes, when paired with properly designed extrinsic motivators.

There has been limited research on implementing gamified behavior simulations in class. Grimani et al. (2024) illustrated that applying game simulation to medication adherence and non‐adherence improved understanding, and consequently, real‐world medication adherence rates. These findings illustrate the gamification potential of diabetes education interventions to improve self‐care and adherence through simulations. When creating gamified interventions, it is important to consider patient preferences. A “one‐size‐fits‐all” strategy could be counterproductive, as Berger and Jung (2024) discovered that individuals have varying preferences for gaming mechanics within diet and health apps. User segmentation can be better understood so that digital interventions are more inclusive and effective. Even though gamification and nudges hold much promise, there remain challenges to overcome. Digital fatigue, disparity in access to technology, and varying levels of health literacy are variables that may influence the impact of these resources (Fleming et al. 2020). Electronic health products require stringent clinical testing and rigorous evaluation of these attributes prior to population‐level gamified behavior‐change interventions to address obesity and diabetes (Gomis‐Pastor et al. 2024).

### 5.2 Use in Diabetes Prevention Programs (e.g., NDPP)

Interventions such as the National Diabetes Prevention Program (NDPP) have begun to incorporate behavior‐change technologies into their integrated efforts towards diabetes prevention. The NDPP is an intervention endorsed by the CDC. Its emphasis lies on behavioral changes, including enhanced physical activity and diet, in a bid to prevent or delay T2D. Gamification strategies have been incorporated into NDPP programs to enhance participation and engagement (Patel et al. 2021).

Social rewards, leaderboards, and points are a few of the strategies employed to maintain individuals' interest in creating positive behavioral changes throughout the program's year‐long operation. A key step in developing an NDPP is the application of nudges. Behavioral nudges, such as reminders of daily activity or coaching sessions, increase program adherence and reduce dropout (Kwan et al. 2020). Today, digital replicas of the NDPP employ app‐based nudge strategies to engage participants, keep them motivated, and track their progress toward their health objectives through numerous touchpoints. Gamification significantly enhances the effectiveness of diabetes prevention programs, says clinical research. Based on a study by Patel et al. (2021), gamified behavior‐based lifestyle change programs resulted in greater weight loss and improved glycemic control than more traditional, non‐digitally interactive interventions.

These findings facilitate the inclusion of gamified strategies within broader public health initiatives, such as the NDPP. AI and ML have revolutionized the provision of personalized therapies as part of diabetes prevention programs. According to Banka et al. (2025), making nutrition counseling and exercise recommendations more personalized with the help of AI algorithm‐generated personalized feedback would render the NDPP more effective and useful for diverse populations. These developments ensure that individuals receive interventions that match their preferences and lifestyles.

These interventions are even stronger when grounded in behavioral economics. By illustrating how clinician choices can be informed by behavioral economics modules within electronic health records, Belli et al. (2020) illustrated how such integration can foster the best preventive care for older adults at risk of diabetes while minimizing overtreatment. One method to enhance risk assessment and intervention tailoring is to apply the same principles to NDPP digital platforms.

However, extensive validation must be performed to ensure that digital treatments are credible in preventative programs. Mathews et al. (2019) emphasized the importance of clinical validation pathways to ensure the security, efficacy, and satisfaction of digital health solution users. Without rigorous validation, computational tools are at risk of being ineffective or even detrimental to the battle against disease. New trials are proving promising, including FOOTSTEPS (Suzuki et al. 2024), a trial that utilizes behavioral economic feedback processes to motivate exercise in individuals at risk for cardiovascular disease and diabetes, two populations with many commonalities. NDPP and similar programs could greatly benefit from what can be learned from such trials. Further support for introducing technology‐enhanced NDPP models was provided through scoping reviews by Kearns et al. (2025), which clearly show the substantial effectiveness of digital technologies that assist in the transfer of mental health and obesity knowledge when integrated with clinical practice. In view of this, mounting consensus now exists that digital health technologies, in the right circumstances, can add to more traditional methods of averting diabetes. Finally, as noted by Gomis‐Pastor et al. (2024), technology‐driven customized coaching, increased integration of gamification with wearable technology, and real‐time analysis of health data are all possible directions for technology to prevent diabetes. Collectively, these elements can create an adaptive prevention environment that enables users to control their metabolic well‐being before disease onset.

## 6 Limitations and Ethical Issues

Although digital health technologies are increasingly being used for diabetes and obesity management, they also present several limitations and ethical issues. The most significant aspect of such issues is data privacy. There is a valid reason to worry about data breaches, unauthorized access, and the secondary use of data without patient consent because of the massive amounts of sensitive health data accumulated by digital tools (Grande et al. 2020). The challenge of ensuring equal protection for everyone is compounded by the lack of equal rules in different regions, fueling these issues. Unfortunately, users must trust that their information is being handled properly. However, if there are any violations or abuses, this trust could be greatly undermined, which would deter individuals from engaging in digital health programs. The high cost of most digital health solutions is also a significant drawback that prevents many from accessing them. Digital treatments can be helpful in treating obesity, but their benefits usually accrue to individuals with a higher socioeconomic status who can afford them, as stated by Hinchliffe et al. (2022). Rather than curtailing health disparities, disadvantaged communities might experience them in enhanced ways because of the exorbitant price of devices such as CGMs, wearable trackers, and subscriptions to apps. For digital health benefits to be universal, we need to eliminate economic barriers. Infrastructure and technical knowledge issues are also included in the accessibility. Kearns et al. (2025) stated that numerous individuals, especially rural dwellers and elderly individuals, struggle to utilize complex health apps properly. User‐unfriendly interfaces, poor Internet connectivity, and insufficient knowledge of mobile technologies also restrict the impact of digital interventions. If these practical factors are not considered, digital health innovations risk failing to reach those who would benefit the most from them. To make digital health available to everyone, developers and legislators must prioritize inclusive design and support services.

**FIGURE 2:**
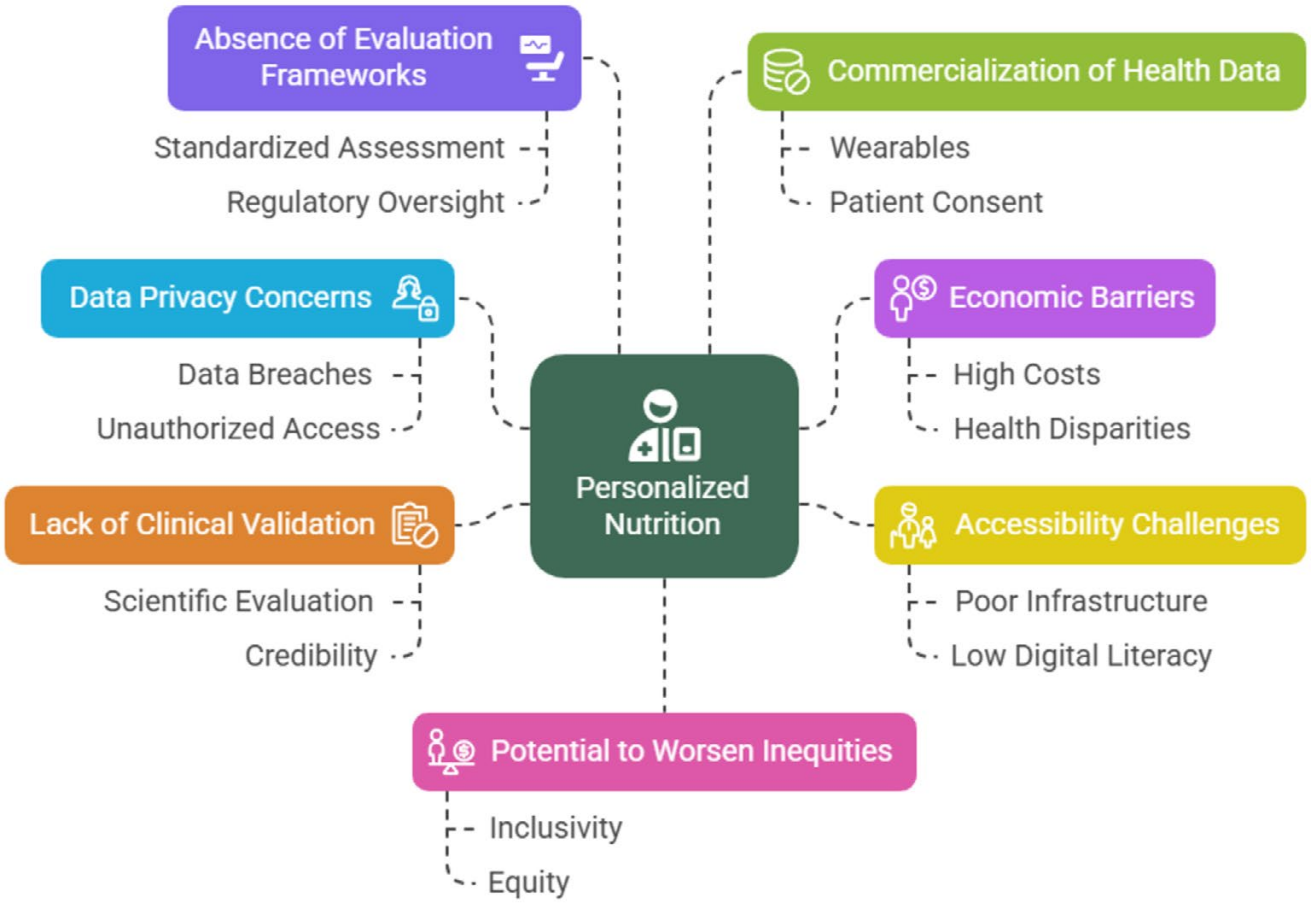
Limitations and ethical issues of personalized nutrition implementation.

Digital health technologies continue to require clinical validation, which is often overlooked even though it is extremely important. Even with the growing digital tool market, various products have not been adequately tested scientifically, raising questions regarding their effectiveness and credibility (Mathews et al. 2019). To ensure the effectiveness of the technologies and to establish the credibility desirable for physician and patient uptake, clinical validation is critical (Gomis‐Pastor et al. 2024). Without confirmation by extensive trials, such technologies risk ending up being called gimmicks instead of effective medical treatments. Another major drawback is that digital health technologies have no established assessment framework. It is difficult to associate, advise on, and control digital tools optimally because of the lopsided health technology assessment, according to a systematic review by von Huben et al. (2022). It is necessary to systematically evaluate safety, effectiveness, and worth, chiefly in the case of treatments that cure chronic conditions such as diabetes and obesity, and this becomes an added concern because there is no clear and evident criterion used for their evaluations. Stringent evaluation standards demand collective efforts from healthcare regulators, practitioners, and technology companies. Commercialization and commodification of health data have ethical consequences. The desire to sell patient data commercially for profit increases as wearable technology becomes more deeply embedded in clinical care (Figure 2) (Ginsburg et al. 2024). Patient choice and well‐versed consent have become less of an issue with the commercialization of intimate health information. To maintain ethical restraints in place as the industry develops, explicit data regulations and patient‐centered governance structures are essential. Finally, it is important to consider the broader context of how the integration of healthcare technology affects society. Notwithstanding the radical potential of digital health technologies, as Solomon and Rudin (2020) put it, their unconsidered adoption risks exacerbating the current inequities. If digital health innovation is going to bring authentic transformation in healthcare, as Bhavnani et al. (2017) state, then it must not let go of the values of presence, equity, and patient‐centeredness. The value of digital health may go unrewarded to many unless active measures are taken to address social and ethical concerns. Figure 2 shows the limitations and ethical issues of personalized nutrition implementation.

### 6.1 Ethical and Equity Considerations in Personalized Nutrition

The intersection of personalized diet with digital health infrastructure catalyzes landmark ethical and equity issues that must be addressed methodically to facilitate real‐world momentum. A key ethical issue is the ownership of data, informed consent, and transparency of algorithms. Given that users enter personal biometric, genomic, and behavioral data through apps and wearable sensors, open governance structures must be in place to prevent misuse and unlawful commodification (Maeckelberghe et al. 2023). Equity concerns are pressing. The application of digital technologies such as CGMs, AI‐driven meal planning apps, and microbiome testing largely preserves more prosperous, urban‐dwelling populations, further widening the digital divide and potentially perpetuating existing health inequities (Paccoud et al. 2024). Marginalized populations, including rural dwellers, elderly citizens, and less digitally informed people, face systemic barriers such as inferior connectivity, ambiguous interfaces, and extortionate pricing plans (Sin et al. 2021). In the interest of justice, subsidized pricing, universal design, and culturally appropriate nutritional guidance are needed. Finally, algorithmic bias trained on small demographic datasets can perpetuate prejudicial diet suggestions. Hence, policymakers and developers must create ethically focused AI frameworks, promote participatory design, and integrate regulatory processes to ensure that personalized nutrition is inclusive and equitable in implementation.

### 6.2 Reimbursement and Regulation Environment

The successful use of digital health tools in personalized nutrition is contingent on clear‐cut regulatory policies and long‐term reimbursement models. However, there are a broad range of differences between geographic markets in evaluating and funding these technologies. The United States Food and Drug Administration (FDA) has provided regulations for software as a medical device (SaMD) that include certain nutrition‐specific digital solutions. However, reimbursement remains patchy, and the majority of nutrition apps and AI platforms are not reimbursable under standard insurance until recommended by healthcare professionals in specific codes (Watson et al. 2023). In contrast, the European Union has adopted the Medical Device Regulation (MDR) model, which deems the majority of AI‐based nutritional aids as Class IIa or greater, necessitating more robust clinical trials. Some EU countries, such as Germany, have also created digital health application (DiGA) pathways where registered apps can become eligible for reimbursement through statutory health insurance if they demonstrate positive health outcomes (Bond et al. 2023).

Regulatory pathways for digital nutrition tools in LMICs are immature. Constraints in infrastructure, a lack of digital health legislation, and unclear approval processes deter innovation adoption. Out‐of‐pocket spending is also dominant in the health financing system, making the reimbursement of digital nutrition interventions non‐existent (Abeltino et al. 2025). To guarantee both scalability and equity, global stakeholders must ensure harmonized standards for the authorization of digital nutrition tools, develop performance‐based reimbursement models, and integrate these tools into primary care systems. Clear regulation and evidence‐based pricing policies will be essential to ensure innovation and access in the new digital world of nutrition.

## 7 Conclusions and Future Perspectives

The use of digital health technology to deliver personalized diets is transforming diabetes and obesity management. Personalized diet interventions involving genetic data, real‐time blood glucose, and AI‐powered feedback can recover metabolic consequences more than unguided recommendations. The use of behavioral interventions as part of an application can make a personalized diet a long‐term strategy for chronic disease management. Data protection, expense, and the need for extensive clinical validation are barriers that need to be addressed. To have a maximum public health impact, it is essential to provide equal access and incorporate these strategies into mainstream medicine. There is increasing evidence that individualized diets can transform strategies for the prevention and treatment of metabolic diseases.

For hyper‐personalized eating advice, the science of personalized nutrition will have to move ahead with multi‐omics integration (genomics, metabolomics, and microbiome) and digital health tools. Augmenting predictive models using AI and ML will enable real‐time adjustment of person‐specific metabolic reactions. Even if regulatory steps are needed to reduce price and availability disparities, wearable technology and mobile health platforms will make it more democratized. Longitudinal research and randomized large‐scale trials are required to validate its efficacy and cost‐effectiveness. Researchers, clinicians, and policymakers must cooperate to standardize procedures and ascertain ethical use. The use of personalized nutrition in public health initiatives could entirely transform the treatment and prevention of metabolic diseases, such as diabetes and obesity.

## Author Contributions

**Muhammad Tayyab Arshad:** writing – original draft (equal). **M. K. M. Ali:** conceptualization (equal), data curation (equal). **Sammra Maqsood:** writing – review and editing (equal). **Ali Ikram:** supervision (equal). **Faiyaz Ahmed:** methodology (equal). **A. I. Aljameel:** data curation (equal), visualization (equal). **Ammar AL‐Farga:** data curation (equal), resources (equal). **Md. Sakhawot Hossain:** validation (equal).

## Disclosure

The authors have nothing to report.

## Ethics Statement

This study did not involve humans or animals.

## Consent

This study did not involve humans.

## Conflicts of Interest

The authors declare no conflicts of interest.

## Data Availability

The data supporting the findings of this study is available from the corresponding author upon reasonable request.
